# Implementation of a Morphological Filter for Removing Spikes from the Epileptic Brain Signals to Improve Identification Ripples

**DOI:** 10.3390/s22197522

**Published:** 2022-10-04

**Authors:** Amir F. Al-Bakri, Radek Martinek, Mariusz Pelc, Jarosław Zygarlicki, Aleksandra Kawala-Sterniuk

**Affiliations:** 1 Department of Biomedical Engineering, College of Engineering, University of Babylon, Hillah 51001, Iraq; 2 Faculty of Electrical Engineering, Automatic Control and Informatics, Opole University of Technology, 45-758 Opole, Poland; 3 Department of Cybernetics and Biomedical Engineering, VSB-Technical University Ostrava—FEECS, 708 00 Ostrava–Poruba, Czech Republic; 4 School of Computing and Mathematical Sciences, University of Greenwich, Park Row, London SE10 9LS, UK

**Keywords:** morphological filter, dynamic threshold, spikes, epilepsy, brain signals, ripples

## Abstract

Epilepsy is a very common disease affecting at least 1% of the population, comprising a number of over 50 million people. As many patients suffer from the drug-resistant version, the number of potential treatment methods is very small. However, since not only the treatment of epilepsy, but also its proper diagnosis or observation of brain signals from recordings are important research areas, in this paper, we address this very problem by developing a reliable technique for removing spikes and sharp transients from the baseline of the brain signal using a morphological filter. This allows much more precise identification of the so-called epileptic zone, which can then be resected, which is one of the methods of epilepsy treatment. We used eight patients with 5 KHz data set and depended upon the Staba 2002 algorithm as a reference to detect the ripples. We found that the average sensitivity and false detection rate of our technique are significant, and they are ∼94% and ∼14%, respectively.

## 1. Introduction

Epilepsy affects over 1% of population, which has been estimated as the number between 50 and 65 million people world wide [1,2,3,4,5,6,7]. Additionally. around 40% of epilepsy-affected patients suffer its drug-resistant version, which significantly limits potential treatment methods [1,2,3,5,8]. It affects the life quality of people affected with it in a significant way, as the seizures occur unexpectedly and can cause various physical injuries or even death [6,9,10,11,12,13,14]. Is is diagnosed usually by analysis of electroencephalography (EEG) signals [6,15,16,17], which can be recorded either from the scalp (surface) or via intracranial EEG (iEEG) [6,18,19,20,21]. The main difference between these two types of recordings is that in the EEG the electrodes are placed on the scalp, and thus, such a procedure is non-invasive, while the intracranial EEG requires surgical intervention as the electrodes are placed directly on the brain exposed surface, which is invasive and risky for patients [6,19,20,21,22].

## 2. Study Background

The iEEG signals can be measured with the use of various implanted electrodes types, such as, among others, standard clinical macro-contacts or special micro-contact [23,24]. Both methods have advantages and disadvantages [21,23,25]. Despite the inconvenience associated with the invasive registration of the iEEG signals, their analysis is a standard procedure in the epilepsy diagnosis [19,21,23,26,27]; it is also due to the quality of the EEG data, which can be characterized with low amplitude spectrum and low frequency ranges, that intracranial recordings can be good alternative to them [25,26,28]. Intracranial recordings can also be used to confirm the information from the signals recorded with the surface EEG [21]. Due to the invasive nature of the iEEG recordings, its use is mainly limited to epilepsy-related studies [26]. It is not a very new technique, as it was invented shortly after classic EEG [25,26,29,30,31]. Contrary to classical EEG, the electrodes are implanted directly into the brain, so local field potentials and spikes can be measured [20,24,27,31,32]. In epileptic patients, the electrodes are implanted for couple of weeks, while patients are hospitalized, in order to record spontaneously occurring seizures [27,31]. Temporal and spatial resolutions of the intracranial EEG are higher than in classical EEG [27,28,31].

Despite significant medicine development and the fact that the past 20 years brought a large number of new anti-epileptic drugs, as mentioned above, only a few treatment methods are destined for epilepsy-suffering patients [1,5]; additionally, epilepsy reduces life expectancy by up to 2 years, particularly in patients affected with cryptogenic or idiopathic epilepsies [5].

As mentioned above, approximately a third of epilepsy patients taking an anti-epileptic drug (AED) may still have seizures, as they are, unfortunately, medication resistant [5,33,34,35,36]. Therefore, the surgical operation to resect epileptic zone is an alternative solution, but, unfortunately, localizing accurately the epileptic zone is sometimes difficult [34,35]. This difficulty is because the seizures usually used to determine the epileptic zone are unpredictable; therefore, this process may need many days or weeks in the hospital to be accomplished [27,31]. Additionally, the surgery is effective in only ca. 62% [1,2,35,37,38]. Each surgery is also a very invasive and risky procedure [1,2,8,37,38,39,40].

Analysis of biomedical data, in particular, brain signals, is a very challenging task [25,31], mostly due to the non-stationary nature of these signals [25]. The EEG signals do not fall into patterns and are inconsistent from one patient to another [31,41]. Additionally, they are prone to various artifact occurrences and vulnerable to noise and/or disturbances, which makes the whole analysis task complicated [31,41].

This study presents a method for using an efficient one-dimensional morphological filter by introducing closing and opening operations on removing spikes and sharp transients from ictal electroencephalography (EEG) signals. Spikes and sharp transients can appear in brain signals due to many reasons, such as physiological features coming along with some brain diseases, for instance, epilepsy, or maybe artifacts [21,22,25,28]. While in some other brain cases, for instance, cognitive task, vision, movement, epilepsy, etc., high frequency true ripples are used as a biomarker [42,43,44,45]. Therefore, in these cases, removing spikes and sharp artifacts, which cause false ripples, is required [46,47]. In fact, initially detecting true ripples using any algorithm required a band-pass filter; therefore, any sharp signal passing through this filter would be presented as a false ripple which is due to the ringing effect of this filter. As a result, these false ripples would affect the outcomes and cause medical misrepresentation [46].

It is possible to differentiate various epilepsy diagnostic methods [48]:

1. Non-invasive (first line):
  - Video EEG;
  - Neuro-psychology;
  - Magnetic resonance imaging (MRI)/functional magnetic resonance imaging (fMRI).
2. Non-invasive (second line):
  - Positron emission tomography (PET);
  - Single photon emission computed tomography (SPECT);
  - Magnetonecephalography (MEG).
3. Invasive (third line):
  - Intracranial EEG.

In patients affected with drug-resistant epilepsy, source imaging (ESI) techniques based on both EEG and ictal EEG are a frequently applied tool [49,50], as it allows automatic zones localization [48,50]. Ictal ESI also allows to provide more accurate scalp interpretation for potential intracranial electrodes placement [48], although it can still be affected by various internal and external artifacts, such as movement, eye blinking, etc. [25,48]. For such reasons, various pre-processing techniques are applied, such as principal component analysis (PCA), independent component analysis (ICA), and filtering [25,48,51].

Instead of conducting a long and time-consuming recording, which would require a very long and expensive stay in the hospital, clinicians try to extract some useful features present in a brief interictal EEG recording [52,53].

It is also important to mention surface ictal EEG, which is one of the non-invasive assessments routinely performed before surgery, used for the purpose of epileptic foci localization [7,54,55,56,57]. It is a reliable and efficient method [56,57,58].

The main aim of any type of epilepsy treatment is to leave the patients seizure free [48,59], regardless of whether it is pharmacological- or surgery based [48,59,60,61,62,63]. As far as surgical procedures are concerned, appropriate epileptic zones localization plays a crucial role [48,59,62].

For such reasons, high frequency ripples have been recently considered as a possible new biomarker for determining the pathological ripple zone that may be used to map the epileptic zone [64,65,66,67]. In fact, there is significant subjectivity in labeling these brief ripples due to false events, and current detection algorithms remain susceptible to common signal spikes and artifacts [65,66,67,68].

A spike is a very short peak presenting in the brain signals, which consists of a peak and a slow wave, which follows immediately after the peak. Typically, an EEG spike is approximately 40–200 ms long [69]. A typical spike is triangular in shape, and it can be distinguished from background activity with an amplitude that is at least twice as high [70]. High frequency oscillations (HFO) or high frequency ripples were recently used as EEG bio markers for epileptic tissues. This feature can be divided into event ripples 80–250 Hz and fast ripples 25–500 Hz [71,72].

The reciprocal inhibition among inhibitory neurons was proposed as a source of ripple oscillations. A physical damage in the inter-neuronal cells causes a pathological issue, which will probably lead to the inhibitory signal reduction upon the pyramidal cells and to the excitatory signal increase.

Finally, the fast ripple oscillations at high frequencies from a pyramidal cell will be arisen [73], where the HFOs were first recorded from intracranial micro-wires [71,74], where the most recent studies have shown that the HFO can be detected from deeper-placed and subdural electrodes [75,76,77,78], but less likely from the surface (scalp) EEG [79,80].

Identifying HFOs by using visual inspection from the ECoG data is a time-consuming, tedious and highly subjective task [81,82,83]. Therefore, Graef et al., 2013 ([84]) suggested an automatic computational technique based on signal detection methodology classification.

Various HFO detection algorithms have already been proposed in the most current literature (see inter alia: [84,85,86,87,88,89]). These simple algorithms used as the first step band-pass filtering and some statistical measurements, such as, among others, RMS (root mean square) [71], line length [87,90] or Hilbert transform [91]. Contrary to the visual inspection, the automated and robust algorithms will take only a short time in order to achieve this task and can save the clinically relevant EEG sections for further applications.

The study presented in [72] and in [75] showed that more than 60% of ripples (80–250 Hz) and about 50% of fast ripples (FRs, 250–500 Hz) occur within spikes. It was also shown that more than 40% of spikes carried ripples and around 30% of spikes co-occurred with fast ripples.

An interesting question can be raised here; how many of the HFOs co-occurring with spikes are true HFOs, and how many are due to the filtering of the sharp transients and were wrongly marked as HFOs? Therefore, there is a need to design and use a reliable, universal and automated software for clinical identification of HFOs. For this purpose, a functional solution should provide an accurate detection of the true HFOs with lowest possible rate of false detection. It should be able to sort out spikes and sharp signals without HFOs. It is difficult to achieve these requirements at the same time due to the trade-off between the sensitivity and false detection rate (FDR) [92].

One of the studies presented by Gliske et al. in 2016 (see: [88,93]), which was based on research carried out by Staba et al., 2002 (see: [71]), described an algorithm developed to identify the HFOs. The aim of their algorithm was to provide an automated, versatile and generalizable method to reject false-positive HFO detections, which appear due to the artifacts. This study showed a reduction in sensitivity for about 10%, but the specificity increased from 68.8% to 88.5%.

For this study purposes, the authors developed a particular technique using a morphological filter that sets a dynamic threshold for removing powerfully spikes and sharp artifacts and improved true ripples detection in the presence of a brief interictal EEG recording. It was done to determine the HFO zone for epileptic patients in order to give additional evidence in defining epileptic zone. After appropriate epileptic area definition, it can be surgically removed, and the patient has a chance to become seizure-free. For that reason, the HFOs have to be properly classified/detected. The problem with that is that the spikes or or sharp transits present in epileptic patients can be detected as false HFOs, particularly when filtered with a band-pass filter of any algorithm used to detect HFOs. Therefore, it is necessary to remove these spikes, thus, this would improve detecting more true HFOs and therefore accurately determine the HFO zone.

## 3. Materials and Methods

For this study purposes, we worked on a method upon boosted, fast and easy spike detection and compensation techniques that will go over the raw signal as a first step and potentially enhance the performance of the automatic HFOs detection algorithm.

### 3.1. Data Selection

Intracranial EEG (iEEG) data were acquired from electrode grids at a high sampling frequency of 5 [kHz], which was selected for analysis from 8 subjects being evaluated for surgical treatment of refractory epilepsy. These data were downloaded from the open source data base, IEEG.org [94]. In order to obtain the events of spikes and ripples, 2 EEG channels located inside epileptic zone marked by physicians were taken for each patient.

Unfortunately, the lengths of the interictal patients data (iEEG) used in this study varied, where the recording lasted 4–24 h.

### 3.2. Study Participants

Table 1 presents the information regarding study participants.

### 3.3. Method for Ripples and Spikes Identification

To more likely achieve some ripples and spikes, random data from interictal bipolar-montage channels placed inside epileptic zone were at first band-pass filtered from 80–500 Hz, and the root-mean-squared (RMS) value in a 3 ms moving window was computed. A sequence of the RMS values that stays above 5 SD (standard deviation) over the mean of the RMS baseline for at least 6 ms was identified as a putative HFO. Events separated by less than 10 ms were clustered together.

An HFO was confirmed to be true if the rectified band-pass filtered signal had 6 or more peaks that crossed a preset threshold (i.e., 3 SD above the mean of a rectified band-pass filtered baseline) [71].

**Figure 1:**
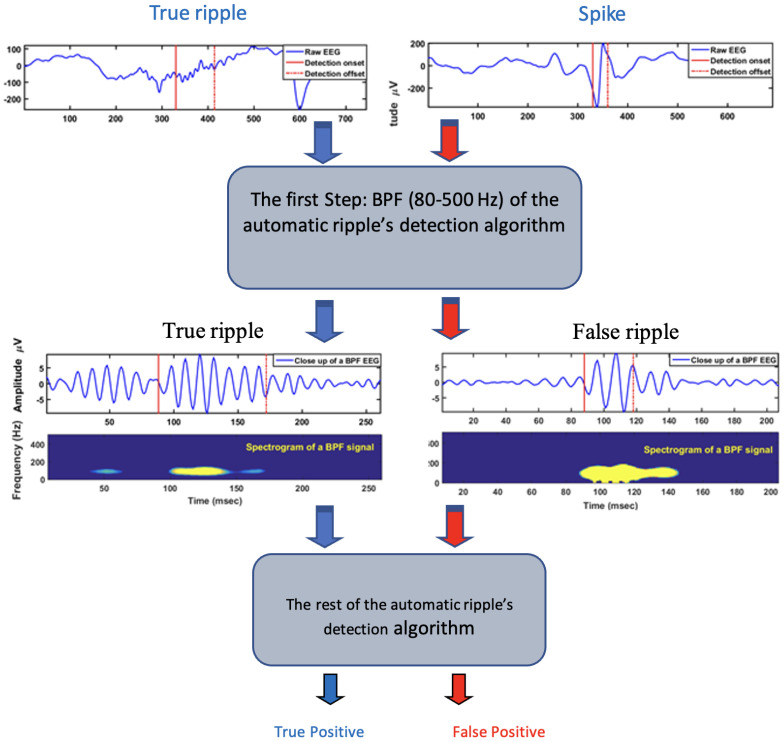
Flowchart—spike detection, true and false positive.

For the study purposes, we coded the Staba 2002 algorithm (see: [71] and applied it for 2 channels randomly recorded data from 8 different patients (4–24 h); as a result, we were able to automatically detect true ripples and, unfortunately, some false positives (due to spikes), as illustrated in Figure 1, where the flowchart shows how the first step (band-pass filtering) of ripple detection algorithm causes false positive results due to spike occurrence (right side).

**Figure 2:**
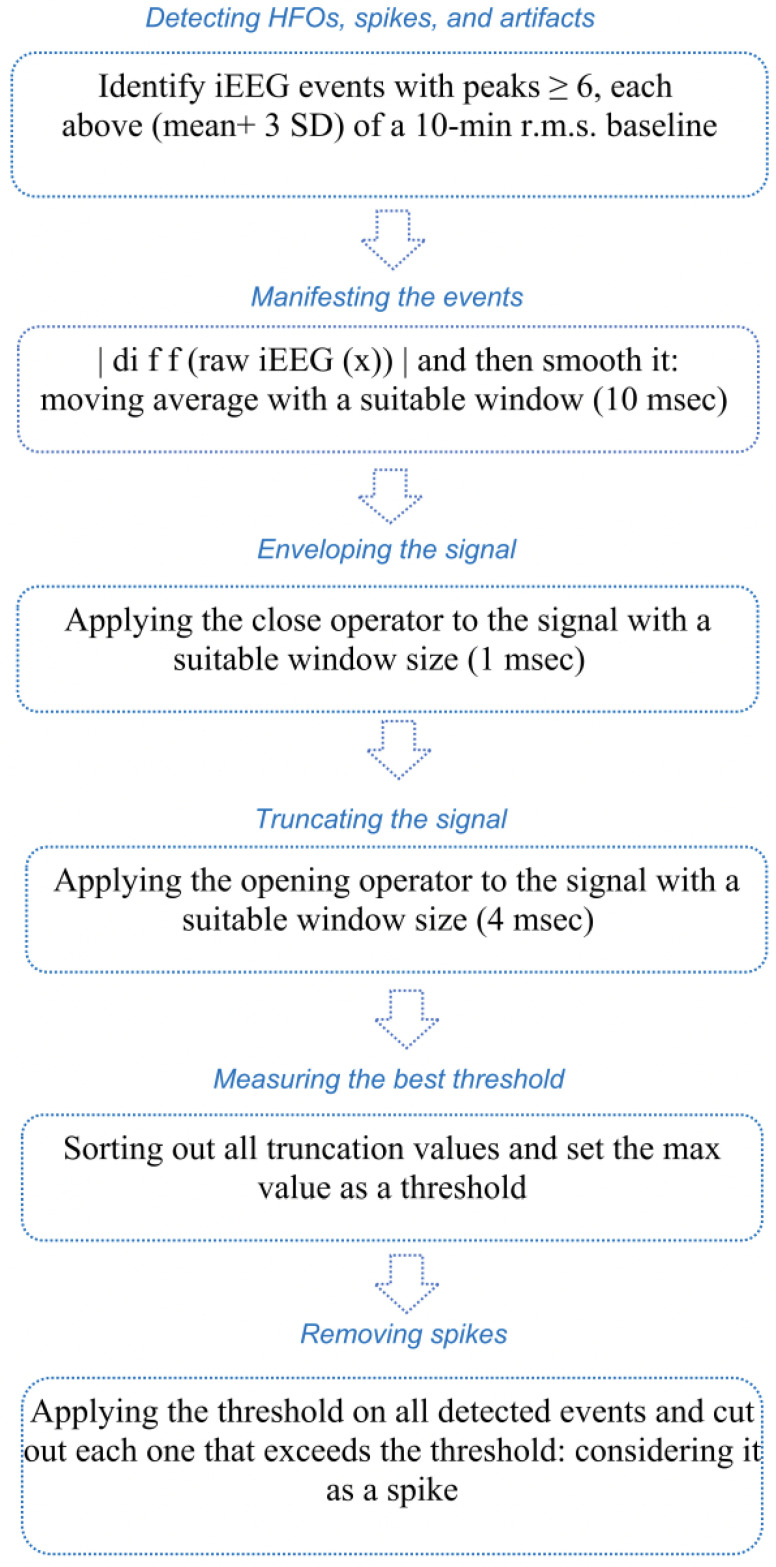
Flowchart with the steps of choosing the best threshold and removing spikes.

Flowchart illustrated with Figure 2 shows the steps taken for choosing the best threshold and for spike removal.

Finally, we used visual inspection to verify the detected ripples and spikes. Here, we considered the detected events as data set and divided into two following groups: training and testing sets. Each set has events of ripples and spikes.

### 3.4. Optimal Threshold for Spikes Truncating Identification

Optimal threshold for spikes truncating identification technique is based on a one-dimension morphological Vanherk filter (closing (Max/Min) and opening (Min/Max)) over the rectified first difference of the raw signal. In this work, we set an appropriate window size of 1 ms and 4 ms for closing and opening operators, respectively.

The purpose for using the morphological filter is to define a suitable threshold in order to distinguish between the background signals and spikes [95,96]. The novelty here is to select a dynamic threshold that depends on the shape of the spikes instead of choosing it by applying an arbitrary fixed threshold over the entire EEG recording. The most morphological filter operations applied here are closing (dilation, then erosion), and opening (erosion and then dilation). The erosion and dilation operations can be framed receptively as (1) and (2) [97]:

(1) $$\left(f⊖g^{s}\right)\left(t\right)=\min_{\tau \in D}\left\{f\left(t\right)-g\left(t-\tau \right)\right)\}$$

(2) $$\left(f⊕g^{s}\right)\left(t\right)=\min_{\tau \in D}\left\{f\left(t\right)+g\left(t-\tau \right)\right)\}$$

Using the above Equations (1) and (2) closing (dilation and erosion), and opening (erosion and dilation) operators can be framed receptively as (3) and (4):

(3) $$\left(f•g\right)\left(t\right)=\left[\left(f⊕g^{s}\right)⊖g\right]\left(t\right)$$

(4) $$\left(f\circ g\right)\left(t\right)=\left[\left(f⊖g^{s}\right)⊕g\right]\left(t\right)$$

where

- f(t)—the analyzed EEG signal;
- g(t)—the structuring element;
- $g^{s}\left(t\right)=g\left(-t\right)$—the reflection of structuring element;
- *D*—the domain of signal f(t).

The one-dimensional operators were described in detail in [98].

**Figure 3:**
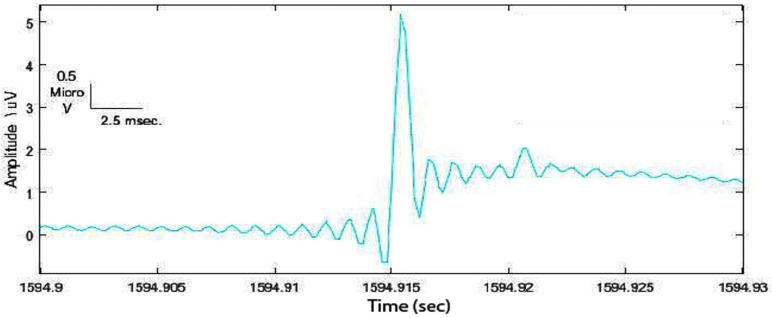
Example of a spike in the data set detected with the Staba 2002 ([71]) algorithm.

**Figure 4:**
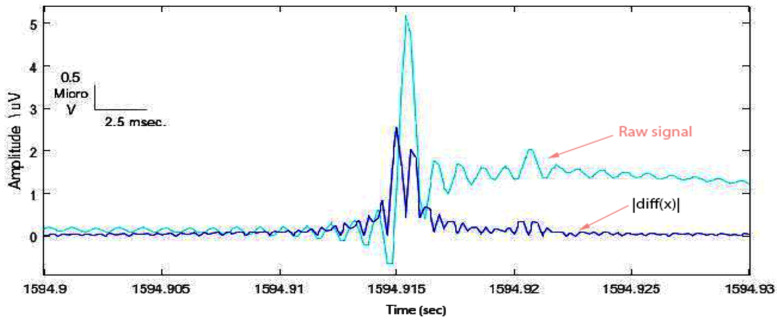
Rectified first difference spike with respect to the original one.

Practically, the steps of applying the one-dimensional morphological filter on iEEG signals in order to find the dynamic optimal threshold are as follows:

**(1)**  Read the raw signal and deal with each event in the data set (Figure 3):

**(2)**  In order to manifest the spike from the EEG background, the rectified first difference signal was computed as ∣diff(x)∣; then the moving average filter with a suitable window size of 10 ms was used to smooth the signal (Figure 4).

**Figure 5:**
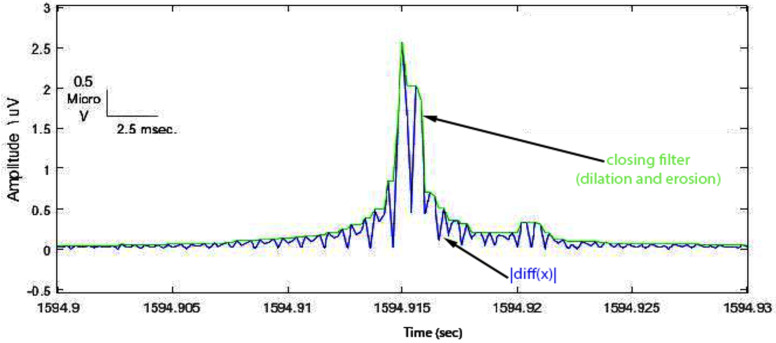
Closing operation demonstrated enveloped spike (green signal).

**Figure 6:**
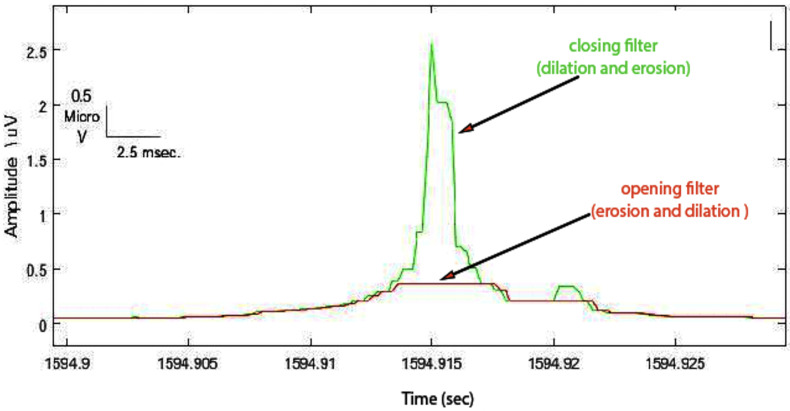
Opening operation demonstrated truncated spike (red signal).

**(3)**  Now it is necessary to apply the one-dimensional morphology filter. The following closing and opening filters were used:
  (a) To envelope the spike and background signal, a closing (dilation, then erosion) filter was applied with an appropriate 1 ms window size (Figure 5).
  (b) To truncate the enveloped spike from an appropriated level, an opening (erosion, then dilation) filter was used with an arbitrary value of 1 ms window size (Figure 6).

**Figure 7:**
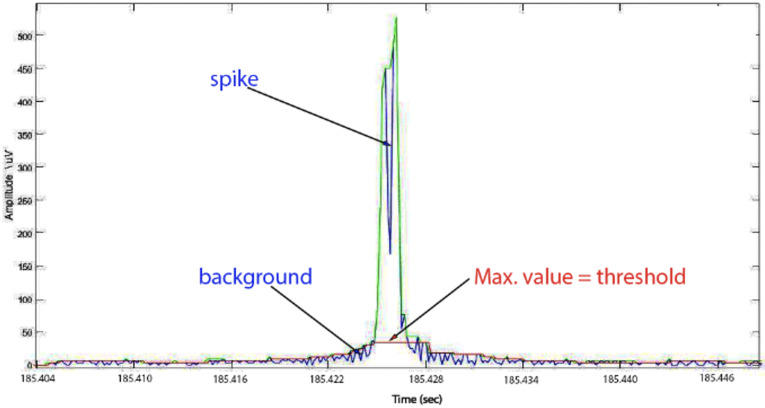
Another spike in the training set with the maximum value of the truncated level. Hint: this value set as the optimal threshold separated between candidate events and background in the data set.

**(4)**  In this step, we sorted out all the truncated values of all events in the training set, then we selected the maximum value to set the initial threshold. As a result, most spikes (false positives) and very few ripples (true positives) were removed from the training set. Now to evaluate the performance of our technique, we measured the sensitivity (SE) and false detection rate (FDR) for all events in the new training set (events of ripples and few spikes) (Figure 7).

However, since the window size of the opening filter is crucial in term of determining the optimal threshold, so instead of choosing it as an arbitrary value (1 ms mentioned earlier), we tried to find a way to identify it. We used the training set and repeated step 4 with different window sizes (1–8) ms and measured SE and FDR for each one. Then, we plotted the receiver operating characteristic curve (ROC) and measured the Euclidean distance between the optimal sensitivity and FDR point (0, 1) (0% FDR, 100% SE), and individual sensitivity and FDR of each window size in order to find the shortest distance and mark the best operation point.

## 4. Results

The open question for applying 1-D morphological filter to remove epileptic spikes from the brain signals was how to determine the suitable window size. In this study, we proposed two operators (closing followed by opening). With the 5 [KHz] sampling rate and very sharp spikes with less than 200 [ms] long window, empirically, we noticed that the window size of the closing operator did not affect too much the smoothing process outcomes. Therefore, we selected an arbitrary 1 [ms] window size and we found that this value was strongly acceptable. From the other hand, we noticed that the big challenge was to set the window size of the opening operator. In fact, this was because the trucking levels that determined the suitable threshold later were so sensitive to the window size. Therefore, we used some statistical measurements, such as SE, FDR and ROC, to achieve and validate the best window size as described below.

After applying the threshold on the data set, the events were classified as listed below:

1. True positive (TP): spikes detected as spikes;
2. False positive (FP): ripples detected as spikes;
3. True negative (TN): ripples detected as ripples;
4. False negative (FN): spikes detected as ripples.

Since we focused on spikes detection, we considered only `TP’, `FP’ and `FN’ in our calculations in order to measure SE and FDR in accordance with the (5) and (6):

(5) SE=TP/(TP+FN)

(6) FDR=FP/(FP+TP)

In Table 2, the nature of events with the spikes’ detection results are presented.

**Figure 8:**
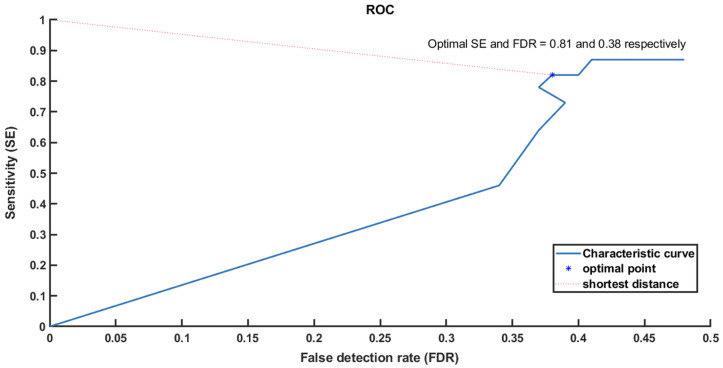
Receiver operating characteristic curve (ROC) shows how to choose the optimal point based on the shortest distance from (0, 1).

From the ROC result (Figure 8), we found that 4 [ms] is the best operation point for these training data sets. We validated this value for the testing data as presented in Table 3.

We repeated the cross-validation technique 10 times for all 10 testing data sets. We found from the ROC results that the window size was consistent at 4 ms. With this filter, we achieved average sensitivity and FDR of ∼94% and ∼14% respectively.

## 5. Discussion and Conclusions

Spikes usually appear in the brain signals for the epileptic patients. They are relatively different in shape within the same patient and across patients and have large and sharp transient areas. Unfortunately, these spikes cause false detection of the biomarker ripples due to the ringing effect of the band-pass filter of any detection algorithm. Therefore, removing them properly from the EEG baseline would improve the detection of high-frequency oscillations (HFOs).

Instead of setting an arbitrary threshold in order to remove these spikes, our hypothesis here is to apply a one-dimensional morphological filter (closing and opening operators) proposed to remove large events. This type of filter would first smooth the EEG signals and second cut out the standout spikes surrounded by lower baseline. In this way, a suitable threshold which is spike shape dependent would be measured and applied to remove as many spikes as possible in the data set.

Interictal spikes and sharp artifacts can be a confounding variable when trying to detect and localize ripple activity. Many algorithms have been proposed to detect spikes which are, in nature, morphologically different. The challenge here is how to set an appropriate threshold while the threshold is shape dependent. Therefore, there is a need to propose a technique which would take into account the shape characteristics in the process. For that reason, we tried to design a reliable spike detection technique by applying a morphological filter. This filter would set a dynamic threshold which is used to catch the most spikes and as a result would improve the detection performance. Generally, our technique is to be used in conjunction with existing automatic ripple detection algorithms.

The verification of ripples is a tedious and subjective process. Improved ripple detection and characterization could help determine the correlation of ripple activity with the epileptic zone in patients being evaluated for surgery.

One of the problems with the data is the number of electrodes placed on the surface of the brain, which depends on how big the area (seizure onset zone) for the investigation is. Additionally, the data used for analysis come from various medical centers, which is another reason for the lack of consistency. 

## Figures and Tables

**Table 1:** Patients’ information.

No.	Subject ID: with 5 [kHZ] Fs	Location	Age	Gender	Data Length	Seizure History	No. of Channels	No. of Seizures
1	I001_P001_D01	Unknown	NA	M	5 days and 4 h	Unknown	62	4
2	I001_P002_D01	Left Temporal Lobe	NA	F	5 days and 9 h	Partial/Complex	15	2
3	I001_P005_D01	Temporal Lobe	NA	M	1 day and 11 h	Partial/Complex	36	1
4	I001_P010_D01	Temporal Lobe	NA	F	4 days	Unknown	56	10
5	I001_P013_D01	Occipital and Parietal Lobes	NA	F	3 days and 13 h	Unknown	72	5
6	I001_P034_D01	Temporal and Frontal Lobes	35	F	1 day and 8 h	Partial/Complex	47	15
7	Study 036	Temporal Lobe	NA	M	4 day and 14 h	Partial/Simple	96	4
8	Study 40	Parietal Lobe	32	M	2 days and 23 h	Partial/Simple/ Complex	116	7

**Table 2:** Part A: describes the nature of the candidate events detected by Staba, 2002 detector. Part B: shows the results of our spike detection technique when we use different window sizes using the training data.

Part A	# of All Candidate Events	# of True Ripples	# of Sharp Transients	# of True Spikes			
	136	113	2	21			
**Part B**	**Window Size** **of the Filter** **[ms]**	**TP**	**FP**	**# of Detectors** **(TP + FP)**	**FN**	**Sensitivity %**	**FDR %**
1	1	9	5	14	12	43	36
2	2	13	7	19	8	62	32
3	3	15	9	24	6	72	38
4	3.4	16	9	25	5	77	36
5	4	17	9	26	4	81	35
6	4.6	17	10	27	4	81	39
7	5	17	11	28	4	81	40
8	5.4	18	12	30	3	86	40
9	6	18	16	34	3	86	47
10	7	18	18	36	3	86	50
11	8	18	19	37	3	86	53

**Table 3:** Part A: describes the nature of the candidate ripples detected by Staba, 2002 detector. Part B: shows the results of our spike detection technique when we used the window size 4 ms for one set of the testing data.

Part A	# of All Candidate Events	# of True Ripples	# of Sharp Transients	# of True Spikes			
	4	2	0	2			
**Part B**	**Window Size** **of the Filter** **[ms]**	**TP**	**FP**	**# of Detectors** **(TP + FP)**	**FN**	**Sensitivity %**	**FDR %**
	4 ms window size	2	2	4	0	100	50

## Data Availability

Not applicable.
